# Retrospective Review on the Use of Topical Cyclosporin A 0.05% for Paediatric Allergic Conjunctivitis in Hong Kong Chinese

**DOI:** 10.1155/2014/396987

**Published:** 2014-10-15

**Authors:** Macy M. S. Wu, Gordon S. K. Yau, Jacky W. Y. Lee, Amy L. Wong, Victor T. Y. Tam, Can Y. F. Yuen

**Affiliations:** Department of Ophthalmology, Caritas Medical Center, 111 Wing Hong Street, Kowloon, Hong Kong

## Abstract

*Purpose*. To evaluate the efficacy of using topical cyclosporin A 0.05% (Restasis) for the treatment of paediatric allergic conjunctivitis. *Methods*. This retrospective study included consecutive cases of paediatric allergic conjunctivitis treated with Restasis between 2010 and 2013. Subjects with follow-up time less than 3 months after using Restasis were excluded. Itch severity score, symptom score, and sign score were compared before (baseline) and 3 months after using Restasis. *Results*. In 27 eyes of 14 patients (mean age 10.8 ± 3.2 years), 44.4% had allergic conjunctivitis, 33.3% had vernal keratoconjunctivitis, and 22.2% had atopic keratoconjunctivitis. The mean duration of ocular symptoms was 20.4 ± 13.2 months. 92.6% of subjects were using steroid eye drop before Restasis. After 3 months of topical Restasis, there were statistically significant reductions in the symptom, sign, and itch severity scores compared with baseline (all *P* ≤ 0.001) and 78.6% of subjects were able to be tapered off steroid eye drops. *Conclusion*. Topical Restasis was effective and safe in significantly reducing ocular itchiness, sign, and symptom scores at 3 months after use in paediatric allergic ocular conditions.

## 1. Introduction

Allergic conjunctivitis is an important disease entity that greatly impacts the quality of life in children as well as the healthcare expenditure [1, 2]. Previous studies have shown a worldwide variation in the prevalence of allergic conjunctivitis and it may be related to ethnic, genetic, and environmental difference [3–6]. There is an increasing prevalence of allergic diseases among Asian children [7].

Hong Kong has a high prevalence of allergic rhinoconjunctivitis. In a large survey conducted in 1997, it was found that 14% of Hong Kong children between 6 and 7 years of age and 24% of 13-14-year olds were affected by allergic rhinoconjunctivitis [6]. Another survey conducted by a local university in 2009 revealed that about 30% of Hong Kong children less than 12 years old were affected by allergic conjunctivitis [8].

Ocular allergies often coexist with allergic rhinitis, asthma, and atopic dermatitis, thus necessitating joint management by ophthalmologists, paediatricians, dermatologists, and immunologists.

Ocular allergies can be broadly classified into seasonal (SAC) or perennial allergic conjunctivitis (PAC), vernal keratoconjunctivitis (VKC), atopic keratoconjunctivitis (AKC), and giant papillary conjunctivitis (GPC). This immune-mediated disorder involves the conjunctiva and, in severe VKC and AKC, corneal involvement may have sight-threatening consequences.

Current treatment aims include (1) relieving symptoms of redness, chemosis, itchiness, blurring of vision, and tearing; (2) limiting the inflammatory process; and (3) preventing damage to the ocular surface. First line treatment often includes avoidance of allergens, cold compress, lubricants, topical antihistamine, nonsteroidal anti-inflammatory drugs, or mast cell stabilizers. Topical steroids are used in patients with more severe and debilitating symptoms. However, steroid drops are notorious for inducing glaucoma and cataract with prolonged usage especially in the paediatric population and some refractory cases may have very limited relief with topical steroids [8]. Oral antihistamine and steroids are sometimes used in severe cases but they are not without side effects.

Restasis (cyclosporin A 0.05%; Allergan Inc., Irvine, CA, USA) is a newer and safer steroid-sparing alternative. Restasis is approved by the United States Food and Drug Administration for the treatment of keratoconjunctivitis sicca or chronic dry eyes. Data on its use in the paediatric population below the age of 16 is lacking [9].

Recently, there have been evidence to support the off-label use of Restasis for allergic conjunctivitis in adult as well as in children, but data pertaining to the Asian population is lacking [10–14].

The aim of this study was to evaluate the efficacy of using Restasis for the treatment of paediatric allergic conjunctivitis in a Chinese population.

## 2. Patients and Methods

A retrospective review of medical records with the diagnosis of allergic conjunctivitis on Restasis treatment was performed at Caritas Medical Centre in Hong Kong, between 2010 and 2013. Cases were eligible for the study if they had unilateral or bilateral allergic conjunctivitis previously treated with at least 3 months of Restasis. Those with incomplete clinical data, allergies to cyclosporin A, or follow-up duration less than 3 months were excluded. Restasis 2 to 4 times daily was added in addition to the subject's topical medication regimen and topical steroids were titrated up or down based on individual clinical response.

The following data were recorded: age, sex, systemic allergic diseases, ophthalmic diagnoses, symptom duration, previous and current medications including steroid use, follow-up duration, and the 3 disease scores: itch severity score (ISS), symptom score, and sign score. The 3 disease scores were recorded before (baseline) and 3 months after using Restasis.

The ISS was adopted which was previously used for the assessment of skin pruritis in our assessment of ocular itchiness. The ISS is a self-reported score with a 0–10 numeric rating scale for itchy symptoms, with 10 being the most severe [15, 16]. The symptom score and sign score measurements were adopted from Ozcan et al. (Table 1) [12].

Normality of the data was confirmed by D'Agostino's *K*-squared test. The paired *t*-test was used to compare allergic ocular disease scores at baseline and 3 months after Restasis. Pearson correlation was used to evaluate the associations between the duration of symptoms and symptom score (baseline and 3 months after treatment), ISS (baseline and 3 months after treatment), and sign score (baseline and 3 months after treatment). *P* value ≤0.05 was considered statistically significant.

## 3. Results 

A total of 27 eyes of 14 subjects were evaluated for this study. The mean age of the subjects was 10.8 ± 3.2 years. The male to female ratio was 13 : 1. The distribution of the allergic ocular conditions was as follows: allergic conjunctivitis (12/27 eyes, 44.4%); VKC (9/27 eyes, 33.3%); and AKC (6/27 eyes, 22.2%). The mean duration of ocular symptoms was 20.4 ± 13.2 months. Allergic systemic associations were present in 8/14 (57.1%) of subjects including eczema, asthma, and allergic rhinitis. All but one patient (92.6%) were using steroid eye drops before start of Restasis (Table 2).

After 3 months of topical Restasis treatment, there were statistically significant reductions in the symptom, sign, and ISS scores compared with baseline (Table 3). Eleven out of our fourteen patients were able to be tapered off steroid drops after instillation of Restasis.

There were no statistically significant correlations between symptom duration and baseline disease scores: symptom score (*P* = 0.7), ISS (*P* = 1.0), or sign score (*P* = 0.2). Likewise, there were no statistically significant correlations between symptom duration and the disease scores at 3 months after treatment: symptom score (*P* = 0.2), ISS (*P* = 1.0), or sign score (*P* = 1.0).

## 4. Discussion

Ocular allergic reaction is a type 1 hypersensitivity reaction with a series of IgE mediated inflammatory reactions involving mast cells, neutrophils, eosinophils, macrophages, and basophils, over the course of hours [17, 18]. Eosinophils can also attract lymphocytes which are more frequently found in chronic atopic disease and may initiate the formation of scars. Atopy is associated with an inherited mutation in the receptor for IL-4 that is associated with enhanced IgE production by B cells and increased T helper cells. The prevalence of allergic conjunctivitis in children with other systemic allergic manifestations can be as high as 15–40% [19].

Cyclosporin is an immunomodulator and it primarily has 2 actions in decreasing inflammation. One is to block cell proliferation and inhibit histamine release from mast cells through inhibition of calcineurin, a phosphate that plays a role in the high-affinity IgE receptor- (Fc*ε*RI-) mediated exocytosis of preformed mediators from mast cells. The second action involves NFAT, a transcriptor regulator for the production of inflammatory cytokines, which is regulated by calcineurin. Therefore, cyclosporin also blocks the release of NFAT-mediated cytokines from T lymphocytes and mast cells, thus reducing eosinophil infiltration and decreasing cellular adhesion to the site of inflammation [12].

Topical cyclosporin has been shown to be effective in the treatment of AKC and VKC but also for allergic conjunctivitis [20], ocular rosacea [21], and refractory seasonal allergic conjunctivitis [22].

Children with VKC have been demonstrated to benefit from Restasis by both a clinical reduction in ocular symptoms and signs and a reduction in inflammatory cell density on impression cytology specimens of the conjunctiva [13]. In our series of Restasis-treated allergic conjunctivitis in a Chinese paediatric population, Restasis significantly reduced both symptoms and signs in allergic, vernal, and atopic conjunctivitis. Meanwhile, 11 out of 14 patients were able to stop using topical steroids, minimizing its potential blinding complications in children. Our findings were consistent with Hingorani et al.'s randomized, placebo-controlled trial of using topical cyclosporin 2% for steroid-dependent AKC. They similarly reported a mean reduction in steroid use together with a reduction in clinical signs and symptoms [10].

The duration of symptoms on presentation showed no statistically significant correlation with disease scores 3 months after treatment, signifying that the drug benefits patients irrespective of their symptom duration and can be useful even in those with prolonged disease. We did not encounter any side effects aside from the transient ocular burning after the application of Restasis which is often the case following most eye drops use. Hingorani et al. used a higher concentration (2%) of cyclosporin and subjects reported intense stinging sensations after use as an indirect result of the diluents used to prepare these eye drops [10]. Akpek et al. and Ozcan et al. later showed that using a lower concentration (0.05%) of cyclosporin resulted in almost no side effects while the efficacy of the drug was still preserved in terms of its steroid-sparing properties in those with AKC [11, 12]. At the time of writing, 12 out of 14 patients were still on Restasis due to the continued beneficial effects.

Our study was limited by the relatively small sample size, single centre, short follow-up, and the lack of a control group. Further randomized-control trials with a larger sample size and longer follow-up duration would provide us with more information about the long-term efficacy of using Restasis for allergic ocular conditions.

Nevertheless, our study demonstrated the clinical usefulness of topical 0.05% cyclosporin eye drops for paediatric allergic ocular conditions without significant side effects.

## Figures and Tables

**Table 1:** Grading of symptoms and signs of allergic conjunctivitis.

Item	0	1	2	3
Symptoms				
Itching	No	Occasional	Frequent	Constant
Tearing	Normal tear	Sensation of fullness of the conjunctival sac	Infrequent spilling of tears over the lid margin	Constant spilling of tears over the lid margin
Discomfort	Absent	Mild	Moderate	Severe
Discharge	No	Small amount of mucoid discharge	Moderate amount of mucoid discharge, presence of crust upon awakening	Eyelids tightly matted together on awakening, warm soaks necessary to clean eyelids during day
Photophobia	No	Mild	Moderate, necessitating dark glasses	Extreme photophobia, even with dark glasses
Signs				
Limbal hypertrophy	No	One quadrant	Two quadrants	Three or more
Bulbar conjunctival hyperemia	Absent	Mild	Moderate	Severe
Tarsal conjunctival papillary hypertrophy	No	Mild	Moderate	Severe, obscuring the visualization of the deep tarsal vessels
Keratitis	No	One quadrant	Two quadrants, macroerosion	Three or more quadrants, vernal ulcer
Neovascularization of cornea	No new vessel formation	Neovascularization in 1 quadrant of cornea	Neovascularization in 2 quadrants of cornea	Neovascularization in 3 or more quadrants of cornea
Cicatrizing conjunctivitis	No	Presence of subepithelial fibrosis	Presence of fornix foreshortening	Symblepharon formation
Blepharitis	No	Mild redness and edema of the eyelid with meibomian gland dysfunction	Moderate inflammation with hyperemia, scales and scurf of eyelid skin	Severe inflammation, with cracks in the eyelid skin, loss of eyelashes, and lid edema

**Table 2:** Case series on the clinical course of allergic conjunctivitis after treatment with Restasis.

Patient	Ocular diagnosis	Age	Sex	Relevant allergies	Symptom onset before use of Restasis	Brief description on disease course	Eye drops in use before start of Restasis
1	LE VKC	6	M	Eczema and asthma	4 weeks	Presented with persistent epithelial defect and cobblestone giant papillae. Developed shield ulcer complicated by herpes simplex infection; ulcer healed with Restasis and acyclovir ointment with only faint corneal scars.	Patanol/ RT/**PF**

2	BE SAC	16	M	Nil	6 weeks	Left limbitis and BE papillae. Treated with Restasis BD, with recovery of the limbitis and improvement of symptoms.	Patanol/RT/**Nadexin**

3	BE AKC	10	M	Eczema and asthma	2 years	Started Restasis BD with improvement of symptoms.	Patanol/RT/**FML**

4	BE VKC	13	M	Allergic rhinitis	2 years	BE cobblestone giant papillae. Started on Restasis BD then tapered down to once daily on alternate days.	Patanol/ RT/**FML**/**PF**

5	BE PAC	12	F	Nil	1 year	Improvement in symptoms and decreased punctuate epithelial erosions and reduced conjunctival injection over the papillae with Restasis BD.	Patanol/**FML**

6	BE VKC	9	M	Nil	2 years	On and off limbitis and papillae. Used Restasis BD for 1 year with good control and 1 flare-up requiring QID. Now down to once daily dose on alternate days.	Patanol/RT/**FML**/**PF**

7	BE AKC	8	M	Eczema and asthma	2 years	BE giant papillae, with reduced conjunctival injection over the papillae Restasis BD	Patanol/RT/**Nadexin**/**Lotemax**

8	BE VKC	10	M	Nil	1.5 years	Giant papillae, with reduced conjunctival injection over the papillae with Restasis BD.	Patanol/**FML**

9	BE PAC	14	M	Asthma	3 years	Previously started with Restasis BD to TDS. Tapered down to once daily on alternate days after 5 months, with improvement of symptoms.	Patanol **Lotemax**

10	BE PAC	10	M	Allergic rhinitis	3 years	Reduced conjunctival injection over the papillae while on Restasis BD.	Patanol/**FML**

11	BE PAC	6	M	Allergic rhinitis	1 year	Reduced conjunctival injection over the papillae while on Restasis BD.	Patanol

12	BE AKC	16	M	Allergic rhinitis	16 months	Papillae with keratitis. Restasis BD used for 4 months, now down to once daily on alternate days, with recovery of the keratitis and improvement of symptoms.	Patanol/RT/**FML**

13	BE PAC	9	M	Nil	4 years	Reduced conjunctival injection over the papillae while on Restasis BD, later stopped due to financial reason.	Patanol/RT **FML**

14	BE VKC	12	M	Allergic rhinitis	2 months	Trantas dots and limbitis with papillae, now on Restasis BD, with recovery of the limbitis and improvement of symptoms.	Patanol/**PF**

BE = both eyes.

LE = left eye.

BD = twice daily.

TDS = three times daily.

Patanol = olopatadine hydrochloride ophthalmic solution 0.1%; Alcon Laboratories Inc., Fort Worth, Texas, USA.

RT = Refresh Tears (carboxymethylcellulose sodium 0.5%; Allergan Inc., Irvine, CA, USA).

PF = Pred Forte (prednisolone acetate 1%; Allergan Inc., Irvine, CA USA).

FML = fluorometholone (Allergan Inc., Irvine, CA, USA).

Nadexin = (dexamethasone 0.1% + chloramphenicol 0.5%; National Pharm Co. Ltd., Unit A-D 7/F Candy Novelty House 164 Wai Yip Street, Kowloon, Hong Kong).

Lotemax = (loteprednol etabonate ophthalmic 0.5%; Bausch & Lomb Inc., Tampa, Florida, USA).

**Table 3:** Disease scores at baseline versus 3 months after Restasis.

	Baseline	3 months	*P* value
Symptom score (out of 15)	8.2 ± 1.3	5.1 ± 0.8	<0.0001∗
Itch severity score (out of 10)	7.9 ± 0.7	4.5 ± 1.0	<0.0001∗
Sign score (out of 21)	7.1 ± 1.5	4.9 ± 0.8	<0.0001∗

^*^Statistically significant.
